# An Incremental Target-Adapted Strategy for Active Geometric Calibration of Projector-Camera Systems

**DOI:** 10.3390/s130202664

**Published:** 2013-02-22

**Authors:** Chia-Yen Chen, Hsiang-Jen Chien

**Affiliations:** Department of Computer Science and Information Engineering, National University of Kaohsiung, 700, Kaohsiung University Rd. Nan Tzu Dist., Kaohsiung 811, Taiwan; E-Mails: ayen@nuk.edu.tw (C.-Y.C.); johnny@jjen.cc (H.-J.C.); Tel.: +886-7-591-9710; Fax: +886-7-591-9514

**Keywords:** projector-camera calibration, structured light, homography, closed-loop system

## Abstract

The calibration of a projector-camera system is an essential step toward accurate 3-D measurement and environment-aware data projection applications, such as augmented reality. In this paper we present a two-stage easy-to-deploy strategy for robust calibration of both intrinsic and extrinsic parameters of a projector. Two key components of the system are the automatic generation of projected light patterns and the incremental calibration process. Based on the incremental strategy, the calibration process first establishes a set of initial parameters, and then it upgrades these parameters incrementally using the projection and captured images of dynamically-generated calibration patterns. The scene-driven light patterns allow the system to adapt itself to the pose of the calibration target, such that the difficulty in feature detection is greatly lowered. The strategy forms a closed-loop system that performs self-correction as more and more observations become available. Compared to the conventional method, which requires a time-consuming process for the acquisition of dense pixel correspondences, the proposed method deploys a homography-based coordinate computation, allowing the calibration time to be dramatically reduced. The experimental results indicate that an improvement of 70% in reprojection errors is achievable and 95% of the calibration time can be saved.

## 1. Introduction

One of the most fundamental problems in the field of computer vision is how to estimate geometric parameters of an image sensor. It forms an active vision system where the image sensor is coupled with a light projector. The performance of such an active vision-based measuring instrument heavily relies on an accurate calibration procedure to determine the geometric parameters of the paired image sensor and light projector. The Microsoft Kinect™ is perhaps one of the most well-known examples [1]. Asides from Kinect's popularity, today's off-the-shelf video projectors are widely adopted to build 3-D scanners due to their cost efficiency and availability [2–4]. Knowing the geometric parameters of a projector also makes it applicable to a wider range of applications, such as augmented reality and performing arts (e.g., [5,6]). The interest in calibrating video projectors has therefore been significantly increasing in the last decade (see [4,5,7–12] for example).

A projector can be effectively described by the pinhole camera model. It is well-known that the geometric parameters of a pinhole camera can be estimated from the world-image correspondences of a set of control points [13,14]. Therefore it is possible to simultaneously calibrate both the camera and the projector using the same object. However, calibrating a projector is not as trivial as calibrating a camera since there is no straightforward way to observe what a projector “sees”, making the establishment of the projector-world correspondences a challenging task.

**Figure 1.**
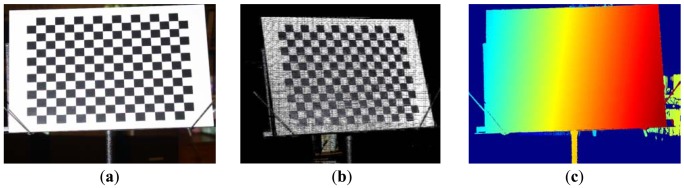
(**a**) A chessboard commonly used to calibration an image sensor. (**b**) Dense reconstruction of the projector's view. (**c**) Visualization of camera-projector *x*-coordinate correspondences acquired using seven Gray-coded patterns and eight phase-shifting patterns.

One approach is to reconstruct the view of the projector from actively acquired camera-projector correspondences (see Figure 1 for example). In order to sample as many control points as possible in the reconstructed view, the process requires establishing dense point-wise mapping from the projection screen to the image plane in sub-pixel precision. It usually involves the projection of a sequence of temporally-codified light patterns, which is not only a time-consuming procedure, but also poses problem when classifying pixels on the stripe boundaries [15]. As a result, dense correspondences come at the cost of either dropped accuracy or increased scanning time, which are not desirable in the calibration process.

**Figure 2.**
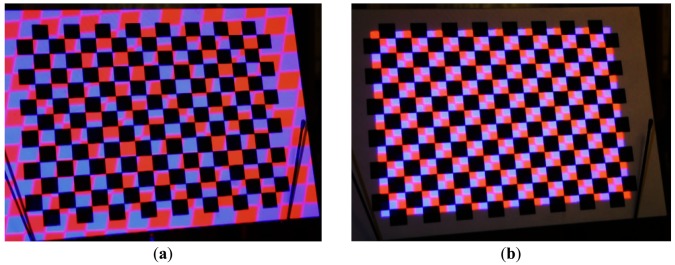
(**a**) Projection of calibration pattern onto a checkerboard. (**b**) The same pattern adaptively aligned to the checkerboard using the proposed method. By taking the geometric displacement into account, the feature points (inner corners) of the two patterns can now be identified distinguishingly.

In past literatures, a typical alternative strategy is to project some easy-to-identify features onto a calibration target (a plane in most cases), which is associated with the so-called world (or global, or object) coordinate system. By analyzing images of the calibration target, a set of projector-world correspondences can be established, and so does the estimation of parameters. One major problem is that, in order to simultaneously identify the world coordinate system and projected features, different types of markers might be mixed together, leading to the interference of patterns [9]. Using a classical checkerboard, for example, could cause difficulty in the detection of marker corners which are projected onto black squares. Figure 2(a) shows that the markers projected onto black squares are barely distinguished.

In this paper, we propose a robust solution to address the aforementioned issues. Observing that markers projected on the white squares of a chessboard are usually clear enough to be detected, we designed an approach to generate aligned calibration patterns so that inference can be avoided, as illustrated in Figure 2(b). The camera-projector correspondences are the prerequisites for the adjustment of patterns. To accelerate the calibration process, we prefer not to use explicit scanning to establish those correspondences. Instead, a one-shot approximation strategy is deployed to estimate the geometric relationships according to previous calibrations. The system is able to use image feedback to examine how the estimated results deviate from actual observation. Once a correction is made, the previously calibrated parameters are refined. Such an incremental calibration strategy starts from rapidly acquired parameters and enhances the accuracy throughout the calibration process as more control points in the space are collected.

The paper is organized as follows: we survey work related to the calibration of camera-projector systems in Section 2. The adopted nonlinear projection model is described in Section 3. An incremental calibration procedure is proposed in Section 4, which is followed by the adaptive generation and detection of dynamically rendered calibration patterns in Section 5. Experimental results are discussed in Section 6. This paper is concluded in Section 7.

## 2. Related Work

The calibration of video projectors has recently received a lot of attention in the field of computer vision. Related work in the literature can be categorized into either photometric or geometric calibration. In this paper we focus on geometric calibration. Since a projector can be described as an inverse camera, many works use the same calibration object to estimate the geometric parameters of the projector while calibrating the camera (e.g., [4,7]). These methods require the acquisition of dense stereo correspondences so that a mapping of control points from projector screen to the world coordinates can be obtained. To achieve better accuracy, the calibration object is replaced many times and the scanning procedure is performed repeatedly. As a result, the calibration time is greatly increased. Experimental results of [4] and [7] show reprojection errors of 0.224 and 0.113 pixels, respectively.

In [8] line patterns are used to find sparse projector-world point correspondences without the projection of sequences of encoded light patterns, achieving a reprojection error of 0.428 pixels. In their work, the projector is assumed to follow the linear projection model. In practice, some projectors may cause non-negligible radial lens distortions, as discovered in our experiments. In this case, the estimated parameters may be far from accurate.

Some methods (e.g., [10,11]) suggest to use another “projector-friendly” object (e.g., a white board) from which the projected calibration patterns are easy to locate. An obvious drawback is that it requires two different targets to calibrate a camera-projector system.

There are also methods utilizing special devices to overcome the interference of calibration pattern. For instance, Zhan *et al.* use a LCD monitor as the calibration target [12]. The panel is turned on with a checkerboard pattern displayed to calibrate a camera and turned off during the projection of light patterns. They have achieved an accuracy of around 0.4 pixels in reprojection error.

## 3. Nonlinear Projection Model and Geometric Calibration

Adopting a model that accurately describes the geometric imaging or projection behavior of a device is critical to the performance of calibration. It has been reported that, like image sensors, an off-the-shelf video projector may pose significant lens distortion due to nonlinear factors which cannot be compensated by the classical pinhole camera model [16]. Therefore, we adopt a modified pinhole camera model with nonlinear correction of radial and tangential lens distortion [13]. Adopting the nonlinear model, a 3-D point (*x, y, z*) expressed in the world coordinate system is first projected onto a point (*u̇*, *v̇*) in the normalized ideal image plane using:

(1) $$\left(\begin{array}{c} \dot{u}\;\\ \dot{v}\;\\ 1 \end{array}\right)\sim \left(\begin{array}{cccc} 1 & 0 & 0 & 0\\ 0 & 1 & 0 & 0\\ 0 & 0 & 1 & 0 \end{array}\right)M_{3\times 4}\left(\begin{array}{c} x\\ y\\ z\\ 1 \end{array}\right)$$

where ∼ means equality up to scale, and:

(2) $$M=\left(\begin{array}{cc} R & t\\ 0 & 1 \end{array}\right)$$

contains the extrinsic parameters that transform points to the camera-centered coordinate system by the rotation matrix *R* ∈ *SO*(3) and the translation 3-vector *t*. The following nonlinear model is then applied to approximate the distorted pixel (*ŭ*, *v̆*) in normalized coordinates:

(3) $$\left(\begin{array}{c} \overset{⌣}{u}\\ \overset{⌣}{v}\\ 1 \end{array}\right)\sim \left(\begin{array}{cccc} \dot{u} & 2\dot{u}\dot{v} & r^{2}+2{\dot{u}}^{2} & 0\\ \dot{v} & r^{2}+2{\dot{v}}^{2} & 2\dot{u}\dot{v} & 0\\ 0 & 0 & 0 & 1 \end{array}\right)\left(\begin{array}{c} 1+\kappa_{1}r^{2}+\kappa_{2}r^{4}+\kappa_{3}r^{6}\\ p_{1}\\ p_{2}\\ 1 \end{array}\right)$$

with the radial term *r*^2^ = *u̇*^2^ + *v̇*^2^ and the distortion coefficients (*κ*_1_, *κ*_2_, *κ*_3_, *p*_1_, *p*_2_). The normalized coordinates (*ŭ*, *v̆*) can be converted to pixel subscripts (*u*, *v*) as:

(4) $$\left(\begin{array}{c} u\\ v\\ 1 \end{array}\right)\sim \left(\begin{array}{ccc} f_{u} & 0 & u_{c}\\ 0 & f_{v} & v_{c}\\ 0 & 0 & 1 \end{array}\right)\left(\begin{array}{c} \overset{⌣}{u}\\ \overset{⌣}{v}\\ 1 \end{array}\right)$$

where *f_u_* and *f_v_* are the effective focal lengths in horizontal and vertical direction respectively, and (*u_c_*, *v_c_*) is the point where the optical axis passes through the image plane. All these parameters plus the distortion coefficients are the intrinsic parameters of a non-linear pinhole camera.

The projection can be denoted by a nonlinear 2-vector function Φ(*x*, *y*, *z*) = (*ϕ_u_*, *ϕ_v_*) parameterized over the intrinsic and extrinsic components. Given a set of world-image point correspondences (*x*, *y*, *z*) → (*u*, *v*) captured from multiple views, one may recover the parameters of Φ. In this work we apply Zhang's calibration method [13] to solve linear parameters (*f_u_*, *f_v_*, *u_c_*, *v_c_*, *M*) first, and then fit the result into the nonlinear model with the distortion coefficients (*κ*_1_, *κ*_2_, *κ*_3_, *p*_1_, *p*_2_) taken into account by minimizing a least-square function in terms of reprojection error. As has been suggested in [14], the reprojection error in horizontal and vertical directions should be dealt with separately, we define the error functions as:

(5) $${∊}_{u}(x,y,z,u,v)=\phi_{u}(x,y,z)-u,\;\text{and}$$

(6) $${∊}_{v}(x,y,z,u,v)=\phi_{v}(x,y,z)-v,$$

and apply an implementation of the Levenberg-Marquardt algorithm to search for the best fitting parameters.

## 4. Incremental Calibration Framework

In this section we present a framework that begins with a few projector-world correspondences and continuously upgrades the estimated parameters of both image sensor and video projector. The proposed calibration procedure works as follows:

1. Several sets of initial world-camera and world-projector correspondences are first collected. This is typically a rapid process using one-shot pattern projection.
2. Initial camera and projector parameters are calculated.
3. An image of the calibration target is captured to calculate its pose.
4. Positions of good feature points that are ideal for projector calibration are calculated using initial parameters and the estimated pose.
5. Pattern renderer generates a calibration pattern according to the calculated positions.
6. Feature points are projected, tracked, and matched to their ideal positions.
7. According to the observed deviation, the projector-world correspondences are updated and the parameters are re-calculated.
8. The process repeats through Steps 3 to 7 until the parameters converge.

As presented above, the underlying algorithms of each step are not limited to any particular method. For example, in Step 1 one may project any pattern that can immediately determine world-projector correspondences. For another example, the implementation may use different types of markers (e.g., circles, squares) to render the projected calibration pattern in Steps 5 and 6.

### 4.1. Framework Overview

**Figure 3.**
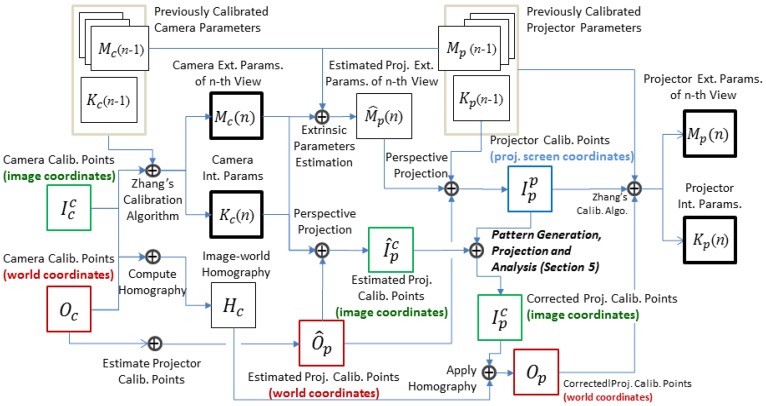
Core components of the proposed incremental calibration method.

The key components of the proposed method are depicted in Figure 3. We first explain the convention of notations used in this paper before going through the process. A calibration point in the 3-D space is denoted by *O*, meaning that it is written in world coordinates. As the devices do not necessarily share the same calibration points, we use subscripts to indicate which device a calibration point is associated with. For example, *O_c_* represents a world point used to calibrate the camera. We use superscripts to further distinguish the projective plane of a calibration point. Symbols *I^c^* and *I^p^* respectively denote a calibration point in the camera's image coordinates and the projector's screen coordinates. Superscripts and subscripts may be used together to specify the purpose and belonging projective plane of a calibration point. For example, ${\text{I}}_{p}^{c}$ denotes the projection of a point used to calibrate the projector in the camera's image plane.

For the *n*-th viewpoint, the procedure starts with an image of a calibration target, from which a set of world-camera correspondences $O_{c}\to {\text{I}}_{c}^{c}$ is derived. This mapping is utilized to update previously calibrated camera parameters *K_c_*(*n* – 1) and *M_c_*(*n* – 1), as well as to compute the homography *H_c_* that transforms pixels to the calibration board. The control points *O_c_* are then taken into account to locate ideal positions *Ô_p_* where the feature points are supposed to be projected to.

Before the formation of a calibration pattern, we need to know which pixels on the projector's screen these points correspond to. This can be done as follows. First the pose of calibration target with respect to the projector is estimated by chaining previously calibrated extrinsic parameters, as will be shown in Section 4.2. Then, *Ô_p_*, the control points in world coordinates are projected onto projector's screen using estimated extrinsic parameters and previously calculated intrinsic parameters, resulting in ${\text{I}}_{p}^{p}$ the locations of feature points on the projection screen.

According to ${\text{I}}_{p}^{p}$, a calibration pattern is rendered and projected onto the scene. Due to the error in the calibrated parameters, *O_p_*, the actual locations of projected features will differ from *Ô_p_*, the estimated locations on the calibration board. We will use image feedback to correct this. To find more accurate correspondences $O_{p}\to {\text{I}}_{p}^{p}$, the feature points are extracted from captured images for further analysis. These points have to be associated with ${\text{I}}_{p}^{p}$ to form calibration data for the projector. The matching can be performed quite efficiently if some hints are available. Hence we use the projection of *Ô_p_* onto the image plane, denoted by ${\hat{I}}_{p}^{c}$, as the starting point of search. Details of the generation and analysis of calibration pattern will be further studied in Section 5.

In the final step, the matched image points ${\text{I}}_{p}^{c}$ are transformed to world coordinates *O_p_* via *H_c_* (see Section 4.3 for the computation and use of homography). Once the world-projector correspondences $O_{p}\to {\text{I}}_{p}^{p}$ are ready, the system performs a multiple-view calibration algorithm which also takes calibration data collected in previous *n* –1 viewpoints to compute refined intrinsic parameters *K_p_* (*n*) and new extrinsic parameters *M_p_* (*n*).

### 4.2. Continuous Calibration and Estimation of Extrinsic Parameters

Collecting calibration data from multiple viewing directions is an important basis to ensure that the calibrated projective parameters can be well generalized to a wide range in 3-D space. The process as described in the previous Section continuously tracks the position of the calibration target and calibrates the devices in real-time. In order to project markers onto specified locations on the calibration target after change of viewing angle, the extrinsic parameters of the projector have to be recalculated. This can be done by solving a classical Perspective-n-Point problem (PnP) given some known 3D-to-2D mapping [17]. However, in our case such world-projector correspondences are not available.

We show that, by chaining previously acquired extrinsic parameters, the rigid transformation from world coordinate system to the projector-centered space can be estimated even without knowing any point correspondences. Let *M_c_*(*i*) and *M_p_*(*i*) be the extrinsic parameters of the camera and of the projector with respect to the *i*-th view, the extrinsic parameters of the projector of the *n*-th view can be estimated using *M_c_*(*n*) and previously calibrated extrinsic parameters as:

(7) $${\hat{M}}_{p}(n)=M_{p}(k)M_{c}^{-1}(k)M_{c}(n)$$

where *k* ∈ {1,2, …, *n* – 1}. Note that the error of previously calibrated parameters will propagate along the chain. Therefore it is important to conduct the calibration and optimization procedures each time a new set of calibration data becomes available.

### 4.3. Projector-World Correspondences from Homography

In our work, the projected feature points are not aligned to the control points printed on the calibration target. As a result, a mechanism is required to assign world coordinates to each projected feature point. The homography from the calibration plane to the image is estimated for this purpose. Under linear projection, the mapping from a pixel (*u*, *v*) to a control point (*x*, *y*, 0) on the calibration plane (*z* = 0) is encapsulated by a homography matrix H as:

(8) $$\left(\begin{array}{c} x\\ y\\ 1 \end{array}\right)\sim {\text{H}}_{3\times 3}\left(\begin{array}{c} u\\ v\\ 1 \end{array}\right)=\left(\begin{array}{ccc} h_{11} & h_{12} & h_{13}\\ h_{21} & h_{22} & h_{23}\\ h_{31} & h_{32} & h_{33} \end{array}\right)\left(\begin{array}{c} u\\ v\\ 1 \end{array}\right)$$

Given at least four point correspondences (*u_i_*, *v_i_*) → (*x_i_*, *y_i_*, 0), the homography can be estimated by solving the over-determined homogeneous linear system [17]:

(9) $$\left(\begin{array}{ccccccccc} u_{1} & v_{1} & 1 & 0 & 0 & 0 & -x_{1}u_{1} & -x_{1}v_{1} & -x_{1}\\ 0 & 0 & 0 & -u_{1} & -v_{1} & -1 & y_{1}u_{1} & y_{1}v_{1} & y_{1}\\ u_{2} & v_{2} & 1 & 0 & 0 & 0 & -x_{2}u_{2} & -x_{2}v_{2} & -x_{2}\\ 0 & 0 & 0 & -u_{2} & -v_{2} & -1 & y_{2}u_{2} & y_{2}v_{2} & y_{2}\\ & & & & \vdots & & & & \end{array}\right)\left(\begin{array}{c} h_{11}\\ h_{12}\\ h_{13}\\ h_{21}\\ h_{22}\\ h_{23}\\ h_{31}\\ h_{32}\\ h_{33} \end{array}\right)=0$$

In this work, the point correspondences are derived from the printed calibration points and their world coordinates (*i.e*., ${\text{I}}_{c}^{c}\to O_{c}$), as shown in Figure 3. Once the homography is estimated, a projected feature point detected at pixel (*u_p_*, *v_p_*) can be associated to its world coordinates according to Equation (8).

In real world scenarios, the homography could be inaccurate when estimated from radially distorted pixels, and in turn, would result in imprecise projector-world correspondences. Therefore it is important to compensate the distortion in advance. To maintain such a camera-projector dependency, the projector-world correspondences will be updated each time the camera's distortion coefficients are adjusted.

### 4.4. Initial Correspondences from Line Patterns

**Figure 4.**
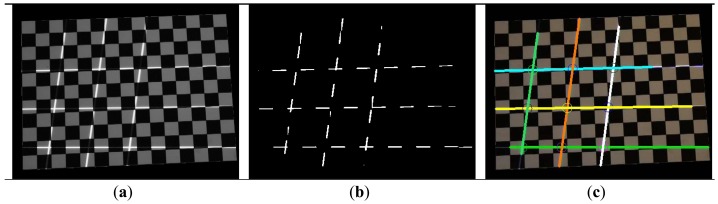
(**a**) Chessboard with the projection of line pattern. (**b**) Detection of the brightest pixels. (**c**) Result of applying the described RANSAC multi-modal line fitting algorithm.

The proposed method requires a “bootstrapping” stage to obtain initial estimate of parameters from which the incremental process can be initiated. In previous work [15], we have used a sequence of colored block patterns that extends the classical 1-D Gray-coded patterns to obtain initial correspondences. In this work, we adopt line features because they are easy and fast to detect, and also more robust against pattern interference and chromatic distortion. Figure 4(a) shows the image of a line pattern projected onto the checkerboard. It is easy to identify six lines despite the fact that some segments of the projected lines are greatly absorbed by the black squares.

A fast technique is designed to reliably locate projected lines. It first searches for the position of the checkerboard in the image using detected corner features. Once found, all pixels in the region of the checkerboard are taken into account to compute a dynamic threshold, such that only the brightest 2% of the pixels survive the binarization (see Figure 4(b) for an example). We then deploy a RANSAC technique to search for lines in the binarized image (see Algorithm 1 for its pseudo-codes). Figure 4(c) shows 9 lines detected in real-time. In the experiments we have found that the adopted method is more robust and faster than Hough Line Transform, which is also a popular line detection algorithm in computer vision. The intersections of six detected lines are then used to establish nine world-projector correspondences and taken into account for the calibration of projector.

**Algorithm 1.** Algorithm of RANSAC-based line detection.
**SearchLinesRansac**
***Input:***
*2-D Points P*, *sampling ratioσ*, *positive ratio of acceptanceρ*, *error tolerance ∊*, *number of iterations k*, *number of lines n*, *point-line distance functionδ****Output:***
*Set of detected planes L*1L ← {∅}2For *i* = 1 to *k*3 Draw a sample *p_i_* ∈ *P* and σ samples *S_i_* ∈ *P* in the vicinity of *p_i_*4 *l_i_* ← *LeastSquareLine*(*S_i_*)5 *P_i_* ←{ *p_i_* ∈ *P:* δ(*p*, *l_i_*) < *∊*}6 If |*P_i_*|/|*P*| > *ρ*: *L* ← *L* ∪{*l_i_*}, *P* ← *P* – *P_i_*,7 If |*L*| = *n*: Exit8End For


## 5. Target-Adapted Calibration Pattern

In this section we describe the generation of the calibration pattern and how to transform the calibration pattern when projecting it onto the target. We also describe the identification of the projected calibration pattern, including how the detected pixels are classified. Measures taken to improve the robustness of the dynamically generated calibration pattern are also discussed.

### 5.1. Pattern Generation

Given a set of true world-projector correspondences *Up: Op* → *Ip*, as well as the projector's intrinsic parameters, *K_p_*, and extrinsic parameters, *M_p_*, that satisfy (*u_p_*, *v_p_*) = Φ(*x_p_*, *y_p_*, *z_p_*), ∀(*x_p_*, *y_p_*, *z_p_*) → (*u_p_*, *v_p_*) ∈ *U_p_*, then the generated pattern, *N*, is expected to contain each feature point located on pixel (*u_p_*, *v_p_*) of projector's screen, such that once back-projected the feature point will project onto the calibration target at point (*x_p_*, *y_p_*, *z_p_*) in world coordinates. Some of the simplest pattern renderings, for example, are to use point features to generate a binary image that contains black background with white dots as can be generated by:

(10) $$N(u,v)=\{\begin{array}{c} 1,\;(u,v)\in I_{p}\\ 0,\;\text{otherwise} \end{array},$$

or with filled circles of radius *r*, given by:

(11) $$N(u,v)=\{\begin{array}{l} 1,\;\exists (u_{p},v_{p})\in I_{p},∥\;(u,v)-(u_{p},v_{p})\;∥\;\leq r\\ 0,\;\text{otherwise} \end{array}.$$

Taking perspective distortion into account, we use corner features in a chessboard pattern. First a base image *B* is rendered in world coordinates, such that each feature is carried on *B*(*x_p_*, *y_p_*). Then the pattern is rendered according to:

(12) $$N(u,v)=B[k_{x}(u,v),k_{y}(u,v)]$$

by means of a mapping function k: (*u*, *v*) → (*x*, *y*). The mapping is essentially a homography transformation applied on undistorted projector screen:

(13) $$k\left(u,v\right)=H_{p}\mu_{K}\left(u,v\right)=H_{p}\left(\begin{array}{c} \dot{u}\\ \dot{v}\\ 1 \end{array}\right)$$

where *μ_K_* (*u*, *v*) =(*u̇*, *v̇*, 1)*^T^* is the inverse function of Equations (3) and (4) defined by intrinsic parameters *K* that reverse the effects of the radial and tangential distortions in normalized image coordinates, and *H_p_* is the homography computed from *μ_K_* (*u_p_*, *v_p_*) → (*x_p_*, *y_p_*, *z_p_*). In the implementation both *B* and *N* are discrete images indexed by integer subscripts while *k* is a real function. Interpolation functions (e.g., bilinear interpolation, as adopted by our work) are used to determine the value of each pixel.

**Figure 5.**
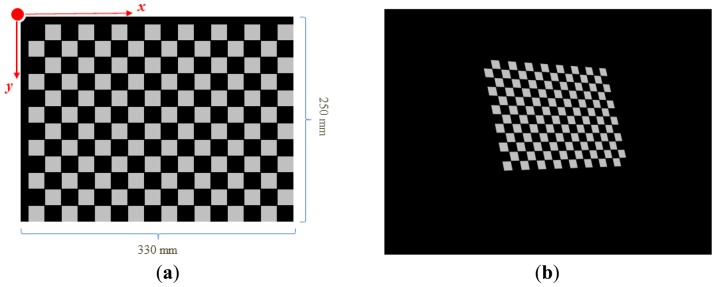
(**a**) A base image annotated with the *x*- and *y*-axis of the world coordinate system. Note the pattern is shifted by 10 mm in both directions, so that the corner features will hit square centers of the calibration target (**b**) A calibration pattern generated from the base image.

**Figure 6.**
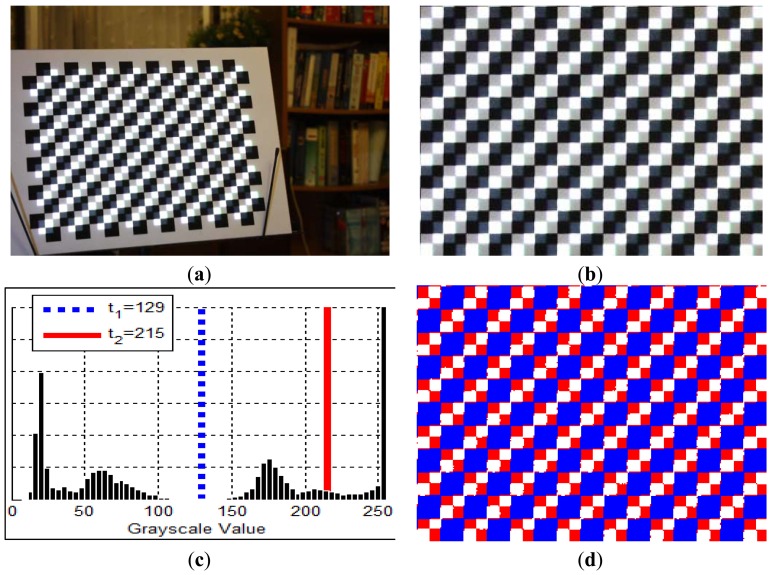
(**a**) Image of the checkerboard with projection of the generated calibration pattern. (**b**) Rectified region of interest. (**c**) Histogram and the optimally determined thresholds. (**d**) Classified pixels, blue, red, and white pixels represent “dark”, “gray” and “bright” categories respectively.

Figure 5(a) illustrates a generated base image which contains corner features aimed to hit square centers of the calibration target—A 16-by-12 checkerboard. Since the squares have dimensions of 20 mm × 20 mm, the pattern is rendered with shifts of 10 mm in both *x* and *y* directions. The base image can be transformed according to Equations (12) and (13) to generate a target-adapted calibration pattern, as shown in Figure 5(b). The projection of this pattern can be found in Figure 6(a).

### 5.2. Marker Detection and Matching in Sub-Pixel Accuracy

**Figure 7.**
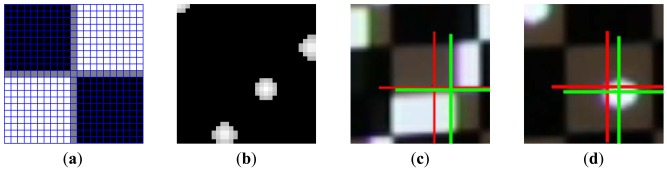
(**a**) Convolution kernel designed to respond corner feature. (**b**) Corner scores computed using convolution (**c**) Crosshairs showing the expected location (in red) and the actual location (in green) of a feature point. (**d**) The same algorithm without convolution can also be used to track spot features.

Image rectification is carried out using camera-world homography *H_c_* (see Section 4.1), then Otsu's threhsolding method [18] is applied twice to categorize rectified pixels into “dark”, “gray”, and “bright” groups. The results are shown in Figure 6. Only “bright” pixels are kept and all others are set to zero. The truncated image is then convoluted with a checker marker as shown in Figure 7(a). The result is normalized to produce corner scores, measuring how likely the corner feature occurs at each pixel. Scores are sorted and filtered so that only the top 5% of the pixels remain. The map is then segmented into regions. For each region a weighhted centroid is calculated to become a candidate feature point.

The feature points detected in the image have to be associated with ${\text{I}}_{p}^{p}$, the rendered feature points on the projector's screen. As aforementioned, the matching can be efficiently done given ${\text{I}}_{p}^{p}\to {\hat{I}}_{p}^{c}$, where ${\hat{I}}_{p}^{c}$ are the expected locations of feature points in the image. We start on each expected location of feature points and search for the nearest observation. If the nearest observation is within a tolerable range, then the corresponding world-projector correspondence is updated via ${\text{I}}_{p}^{p}\to {\hat{I}}_{p}^{c}\to {\text{I}}_{p}^{c}$. Otherwise the feature point is marked as lost, and will be absent in the refined calibration data. Figure 7(c) shows a pair of matched ${\hat{I}}_{p}^{c}$ and ${\text{I}}_{p}^{c}$. The centroid extraction can also be applied to locate a spot features as shown in Figure 7(d).

### 5.3. Rejection of Calibration Data

A dynamically generated calibration pattern can miss its target after projection, if previously calibrated parameters have failed to be generalized to the new pose of the calibration board. It usually occurs when the system has not collected enough calibration data and the board has changed to a pose that is significantly different from its previous geometric configuration. It is necessary to detect such situation to prevent wrongly associated correspondences being used as valid calibration data.

The failure of a generated calibration pattern occurs if there are too few matched feature points, *i.e.*, the observed results deviates greatly from our expectation. Hence, we set a condition to reject a calibration pattern if more than 50% of the feature points are lost. The generalization error is also taken into account to improve the robustness. The newly calibrated parameters are evaluated using each previously collected calibration data. If the inclusion of the new calibration data does not improve the overall performance for more than 50% of the calibrated views, it will be rejected as well.

## 6. Experimental Results

### 6.1. Test Datasets

A projector-camera system has been set up, and a series of experiments have been conducted to evaluate how the proposed method improves the calibration process of a projector-camera system. The hardware specifications are listed in Table 1. The software is implemented on an Intel Core i7 quad-core laptop. The real-time detection of lines and all other computations are not GPU-accelerated. We use a customized checkerboard shown in Figure 6(a) as the calibration target. There are 192 corner features printed on the board, with 83 inner white squares for the projection of calibration feature points. The checkerboard's pose is changed 22 times during the acquisition of calibration data, with the first 4 poses used to generate the initial correspondences as described in Section 4.4.

Two different types of features, namely checker corners and light spots, are used to generate target-adapted calibration patterns (see Figure 7(c)and 7(d) for example). The established calibration datasets are named **AdaCheckers** and **AdaSpots** accordingly. These two datasets are compared with the dataset **Conventional**, which acquires camera-projector correspondences using 14 Gray-coded and 16 phase-shifted patterns for each viewpoint [3].

We have implemented a Levenberg-Marquardt optimizer and applied it to all of the datasets, with identical tuning parameters and termination criteria. The linear and nonlinear parts of the calibrated parameters are listed in Tables 2 and 3 respectively. The calibrated camera parameters are also listed for reference. The asymptotic standard errors are also given in the tables to provide the confidence in parameter estimation. The standard errors are derived from the inverse of the numerically approximated Hessian matrix. The explicit establishment of camera-projector correspondences requires 704 frames to finish the calibration, while the proposed method uses 40 frames, which is only about 5% of the number of frames required by the conventional method. Other results are studied in the rest of this section.

### 6.2. Evaluation in Projective Plane

Reprojection error (RPE) is a commonly adopted projective indicator of how well the calibration data conform to the projection model with the calibrated parameters [7,8,10–14]. We have measured reprojection errors in *x*- and *y*- coordinates separately according to Equations 5 and 6, and the root-mean-squares (RMSs) are calculated to summarize the performance of the parameters with respect to a dataset.

**Figure 8.**
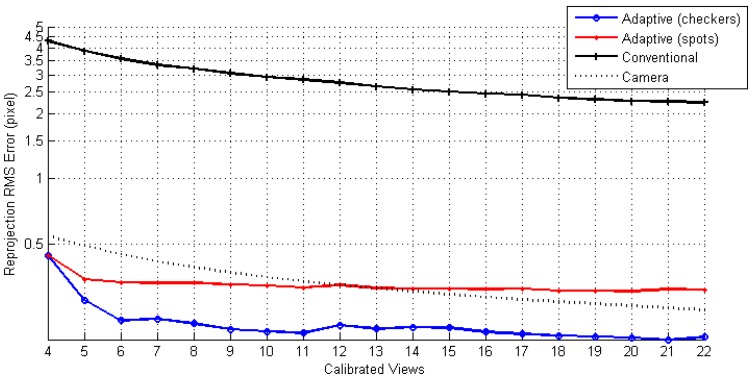
History RMS of reprojection errors during the calibration process.

After calibrating all 22 views, datasets **AdaCheckers**, **AdaSpots,** and **Conventional** have achieved RPEs of 0.188, 0.301, and 2.196 pixels respectively. Figure 8 depicts their RPEs at each stage, with a set of calibration data collected from a new viewpoint. One may find that the adaptively rendered calibration patterns initially pose significant errors. However, the errors decrease as more calibration data are collected with a lower bound. The RPEs of the **AdaSpots** are 1.6 times higher than that of the **AdaCheckers**. The cause might be that the algorithm has adopted to locate spot centroids, which are not preserved under perspective projection. Compared to the use of explicit correspondences, the adaptively established datasets **AdaCheckers** and **AdaSpots** result in improvements of 91% and 86%, respectively.

### 6.3. Evaluation in Euclidean Space: Planarity Test

Using criteria that are not modeled in the objective functions is important to evaluate the optimized parameters. We have therefore conducted another test to verify the performance of the calibrated parameters in 3-D space. The parameters are used to triangulate the 3-D position of each control point. Since a planar target is used, all of the control points are expected to lie in a plane. The flatness of the manufactured calibration target has been assessed to be accurate to within 0.1 mm.

**Figure 9.**
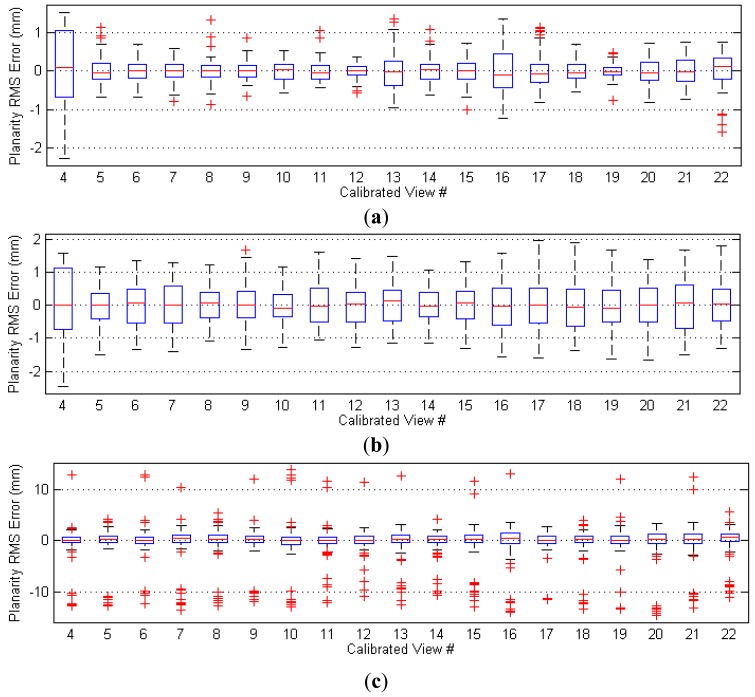
Plane residuals of control points after triangulated using parameters calibrated from datasets (**a**) **AdaCheckers** (**b**) **AdaSpots**, and (**c**) **Conventional**.

The best-fit planes of measured 3-D points are estimated, and the residuals are calculated. The box plots of the residuals are depicted in Figure 9. The overall 3-D RMS errors for the three datasets are 0.36 mm, 0.65 mm, and 2.42 mm, respectively. Improvements of 85% and 73% are achieved for **AdaCheckers** and **AdaSpots**, respectively.

### 6.4. Evaluation in Euclidean Space: Triangulation Error

The projector-camera system can be utilized as a structured light 3-D scanner once the extrinsic and intrinsic parameters of the camera and the projector are obtained. Given a dense correspondence map, one may apply triangulation to recover the surface of an object. The triangulation error is defined as the shortest Euclidean distance between a pair of back-projected rays. It can be used to evaluate the calibration, since a more accurate set of parameters implies that the back-projected rays are more precise, and consequently, the triangulation errors will be lower.

**Figure 10.**
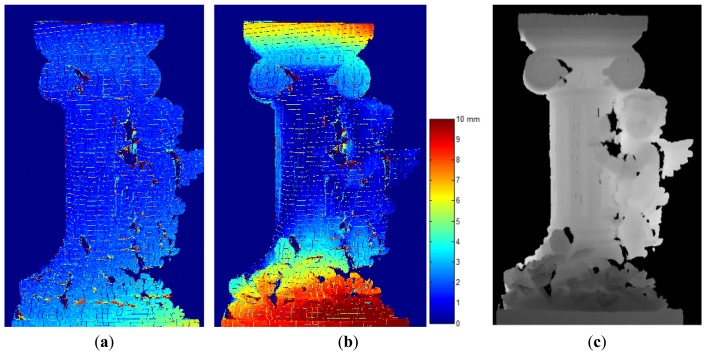
Triangulation error map of the parameters calibrated using datasets (**a**) **AdaCheckers** and (**b**) **Conventional**; (**c**) Recovered depth map of a scanned statue.

A statue with a highly irregular shape is selected to be scanned by the projector-camera system. The statue has a dimension of 450 mm by 250 mm by 240 mm in height, width, and depth. The statue is placed inside a working space spanned by the positions of the calibration board. About 105,000 points are triangulated using parameters calibrated from the **AdaCheckers** and **Conventional** datasets, and the calculated RMS errors are respectively 2.71 mm and 5.10 mm, or 1.8% and 3.4% compared to the depth of the recovered surface (145 mm). The error maps are shown in Figures 10(a) and (b).

There are observable systematic errors in the recovered surface using parameters calibrated from the **Conventional** dataset when the error maps are compared to the depth map shown in Figure 10(c). This is a commonly observed phenomenon if a set of parameters is not well generalized to the overall volume. As a measured point moves away from the optimal space of the parameters, the triangulation errors will increase quadratically. Based on this observation, we may verify that the parameters calibrated from the **AdaMarkers** dataset are more robust since they conform better in a wider range.

## 7. Conclusions and Future Work

In this paper, we have presented an innovative method to reliably establish the calibration datasets for a project-camera system. The method can be applied to achieve the calibration of the camera and the projector using as the calibration target a single checkerboard, which is easy to obtain and widely used in the computer vision. The dynamically generated calibration patterns contain feature points for the calibration of the projector. Each feature point is arranged to hit the center of a particular white square on the calibration target, where its detection can be accurately performed. With a feedback mechanism, the system increases the accuracy of the generated patterns incrementally. As a result, establishing a calibration dataset becomes more accurate and faster than deploying dense acquisition of camera-projector correspondences. In the experimental results, the proposed method is able to achieve an improvement more than 80% over the conventional method in both projective and Euclidean tests, with a saving of 95% of the required calibration time. The RPEs in sub-pixel level are also attainable.

In the future, we aim to develop a system that tracks calibration targets and projects aligned calibration patterns in real-time. As can be seen in Figure 6, the corner features printed on the calibration target are still distinguishable due to the projection of the interleaved calibration pattern. Such a real-time application may hint a user to move the calibration target toward un-sampled space, instead of placing it randomly; since it is crucial to maximize the coverage of the working volume during the collection of calibration data to achieve accurate 3-D measurement,. The proposed method can also be modified and adapted towards the application of environment-aware data projections, such as those used in augmented reality applications.

## Figures and Tables

**Table 1.** Hardware specification in the setup of the project-camera system.

**Component**	**Model**	**Image Resolution**	**Lens Parameters**	**Data Rate**
**Camera**	Canon EOS 450D (EVF Stream Mode)	848 × 560 pixels	18—55 mm (F3.5—F5.6)	12 FPS
**Projector**	Epson EB-X7	1,024 × 768 pixels	16.90—20.28 mm (F1.58—F1.72)	60 FPS

**Table 2.** Calibrated linear intrinsic parameters with standard errors.

**Calibration Dataset**	***f_u_***	***f_v_***	***u_c_***	***v_c_***
**AdaCheckers**	1,588.1 ± 36 pixels	1,560.7 ± 35 pixels	561.6 ± 19 pixels	562.7 ± 29 pixels
**AdaSpots**	1,619.7 ± 38 pixels	1,605.0 ± 41 pixels	535.0 ± 39 pixels	590.8 ± 27 pixels
**Conventional**	1,812.1 ± 23 pixels	1,686.1 ± 20 pixels	287.5 ± 10 pixels	530.2 ± 06 pixels
**Camera**	1,273.1 ± 14 pixels	1,258.0 ± 13 pixels	433.4 ± 08 pixels	274.4 ± 07 pixels

**Table 3.** Calibrated nonlinear intrinsic parameters with standard errors.

**Calibration Dataset**	***k*_1_**	***k*_2_**	***k*_3_**	***p*_1_**	***p*_1_**
**AdaCheckers**	0.577 ± 0.15	−9.288 ± 4.70	65.034 ± 46.5	0.009 ± 0.01	0.024 ± 0.01
**AdaSpots**	0.146 ± 0.01	−0.892 ± 3.17	2.878 ± 27.8	0.007 ± 0.01	0.004 ± 0.01
**Conventional**	−1.332 ± 0.07	13.851 ± 1.71	−62.128 ±11.7	−0.006 ± 0.00	0.033 ± 0.00
**Camera**	−0.089 ±0.05	1.370 ± 1.33	−6.841 ± 10.9	−0.003 ± 0.00	−0.002 ± 0.00
